# Improved Adherence to Vision Self-monitoring with the Vision and Memory Stimulating (VMS) Journal for Non-neovascular Age-related Macular Degeneration during a Randomized Controlled Trial

**DOI:** 10.4172/2155-9570.1000320

**Published:** 2014-01-22

**Authors:** Ava K Bittner, Sheryl Torr-Brown, Ellen Arnold, Antonia Nwankwo, Patricia Beaton, Radhika Rampat, Gislin Dagnelie, Mark Roser

**Affiliations:** 1 Johns Hopkins Wilmer Eye Institute, 600 N. Wolfe St., Baltimore, MD 21287, USA; 2 KeepSight, 515 Old Slocum Rd, Hebron, CT 06248, USA; 3 Moorefields Eye Hospital, London, UK; 4 Nova Southeastern University, College of Optometry, 3200 South University Dr., Ft. Lauderdale, FL, 33328-2018, USA

**Keywords:** Age-related macular degeneration, Vision, Self-monitoring, Amsler grid, Adherence

## Abstract

**Objective:**

An educational, interactive journal [Vision and Memory Stimulating (VMS) journal] was developed to boost patient confidence and promote long-term adherence with weekly vision self-monitoring in age-related macular degeneration (AMD) patients at risk for vision loss from new-onset neovascularization.

**Methods:**

In a multicenter randomized controlled trial, 198 subjects with intermediate stage, non-neovascular AMD received the VMS journal or followed usual care (e.g. their doctor’s instructions for vision monitoring; Amsler grid). At 6 and/or 12 months post-enrollment, 157 subjects completed a questionnaire on vision self-monitoring.

**Results:**

At 6 and 12 months, respectively, 85% and 80% of the VMS journal subjects reported vision monitoring at least weekly, which represent statistically significant 7.1 and 4.2 times greater odds than the 50% of controls who monitored weekly at both follow-up times (p<0.001). At 6 and 12 months, respectively, 29% and 25% of controls indicated that they had not checked their vision in the past 6 months, while only 1.5% and 5% of the VMS journal subjects reported no vision self-monitoring. At 6 and 12 months, respectively, only 15% and 13% of the VMS journal subjects vs. 53% and 44% of the controls reported that they did not feel confident that they were taking care of their sight by self-monitoring (p<0.001). Usual care controls had statistically significant 6.7 and 5.0 times greater odds of reporting non-confidence at 6 and 12 months, respectively. There was no statistically significant change in weekly vs. less frequent self-monitoring between the groups (p=0.68), with 81% of all subjects reporting no change in frequency between 6 and 12 months.

**Conclusions:**

These findings support the efficacy of the VMS journal for increasing vision self-monitoring adherence and confidence, in addition to promoting persistence in weekly monitoring over the course of a year in AMD subjects at risk for exudative retinal changes.

## Introduction

Interest in remote, home-based monitoring of chronic diseases is greatly increasing, especially for diseases in which early intervention improves patient outcomes. One example is the neovascular form of age-related macular degeneration (AMD), for which treatment can halt or partially improve vision loss if not delayed. Vision loss from new onset, neovascular disease occurs rapidly and unexpectedly, but is often undetected and has a worse prognosis if not treated immediately [1,2]. Thus AMD continues to be the leading cause of vision loss in those over age 60, primarily because many patients wait until they have suffered significant vision loss before making a decision to seek help. In order to optimize clinical outcomes, patients must take the initiative to routinely self-monitor their vision and present promptly upon new symptoms of vision loss. Seeking timely evaluation and treatment would help reduce the risk of damage to the fovea from neovascularization, which expands across the retina at 25 microns/day on average [3].

Delays in presentation to an eye care provider following the onset of neovascular AMD are attributable to a variety of factors, including lack of adherence with vision self-monitoring regimens, lack of confidence in recognizing symptoms of vision loss, and attributing vision loss to non-urgent or non-treatable causes due to the lack of awareness about AMD types, stages, risks and proper self-management strategies. The typical delay in presentation to an eye care professional following the development of neovascular AMD has been estimated as approximately 5 months based on typical progression of lesions over time [4]. Delay in the start of anti-VEGF treatment by several months (i.e. >21 wks. vs. <7 wks. after an exudative event) has been shown to be a significant risk factor for worse vision outcomes [5]; therefore, it is a critical health care challenge to improve patients’ vision self-monitoring tools so that they address and overcome the various reasons for patients’ delay in seeking evaluation and treatment.

The current standard of care for AMD self-monitoring is the Amsler grid, which has a poor track record for detecting vision changes due to early neovascular AMD [6–8]. In previous studies, the Amsler grid detected vision abnormalities in <30% of patients who subsequently required treatment for neovascular AMD [6] and failed to detect the majority of standard and threshold scotomas (77% and 87%, respectively) [7]. One study revealed variability in the size, shape and location of scotomas when two Amsler tests were successively administered [8]. Thus, the suboptimal performance of the Amsler grid for detecting new-onset neovascularization may be related to multiple factors, such as perceptual completion phenomena, inconsistent fixation and/or poor compliance with regular monitoring by patients. More importantly, even when symptoms are observed when using the Amsler grid, a significant number of patients fail to self-refer, highlighting the need for a more holistic approach in the design of home monitoring solutions that address a broader scope of reasons for delay. Despite its drawbacks, the wide distribution of the Amsler grid is noteworthy since it demonstrates the feasibility of distributing low-tech, low-cost tools to very broad, large scale patient populations.

AMD patients may experience various initial symptoms after developing a neovascular lesion, including blurry vision, wavy lines, and/or colored or blank spots. These symptoms can be influenced by filling-in phenomena and can be intermittent, further adding to the patient’s lack of confidence regarding whether a true change in vision has occurred and requires immediate evaluation. Even after patients perceive a symptom as a visual change, they may incorrectly attribute it to non-urgent causes such as cataracts, a need for new glasses or non-neovascular AMD progression, creating another impediment to appropriate self-referral. A recent study showed that awareness of a diagnosis of AMD did not lead to better outcome in terms of visual acuity or lesion size among patients who developed neovascular AMD [9]. Therefore, awareness of the diagnosis alone appears to be inadequate and broader interventions are needed to further educate patients about regular vision self-monitoring and appropriate self-referral when changes in vision are detected. One possible solution to address patients’ lack of knowledge of AMD symptoms and the disease process is to reinforce and supplement the eye care provider’s verbal instructions with a take-home educational journal.

The Vision and Memory Stimulating (VMS) journal was created as a low cost tool with the potential for broad distribution and possible successor of the Amsler grid. It was designed specifically to address three of the top reasons for patient delay in self-referral for treatment following newly developed loss of vision due to neovascular AMD. They are: (1) lack of adherence with self-monitoring regimens; (2) lack of confidence in self-monitoring symptoms of vision loss; and (3) lack of awareness of the disease, its risks and proper self care methods.

The VMS journal was designed through extensive AMD patient engagement, direct observation and feedback from AMD patients. It is intended to be used once each week to augment daily observations. The journal is presented in a large font, journal format with two new pages to complete every week and stickers to track completion. To overcome lack of adherence, the system incorporates a reminder-based journal approach to boost engagement and offers game-based visual acuity testing, as a vehicle to drive patient engagement. The VMS journal also provides weekly inspirational sayings and a pleasant graphical design, based upon preferences identified during AMD patient focus group interviews. To overcome lack of confidence in judging whether new visual symptoms have developed, the system includes multiple vision tests, each with clear instructions, simulated views of possible symptoms and a method for comparing vision against a baseline observation, with the aim that several vision tests would simultaneously confirm and reinforce patients’ observations. To improve lack of awareness of symptoms, understanding of risks, and proper self-care strategies, the system incorporates repetitive, consistent educational messages delivered at a level appropriate for easy cognitive understanding. Lastly, but perhaps most importantly, to promote routine eye exams, the system has a feature to remind patients of follow-up appointment dates with their eye care professional.

A typical educational pamphlet would likely be read once or twice, then discarded and possibly forgotten, while the intent of the VMS journal is to deliver and reinforce consistent, educational messages repeatedly over time (i.e. the need to see a doctor after any perceived change in vision, facts about AMD disease progression, symptoms of vision loss, lifestyle changes to reduce risk of vision loss due to AMD). The VMS journal design promotes weekly use, thereby enabling repeated delivery of these core educational themes.

**Figure 1:**
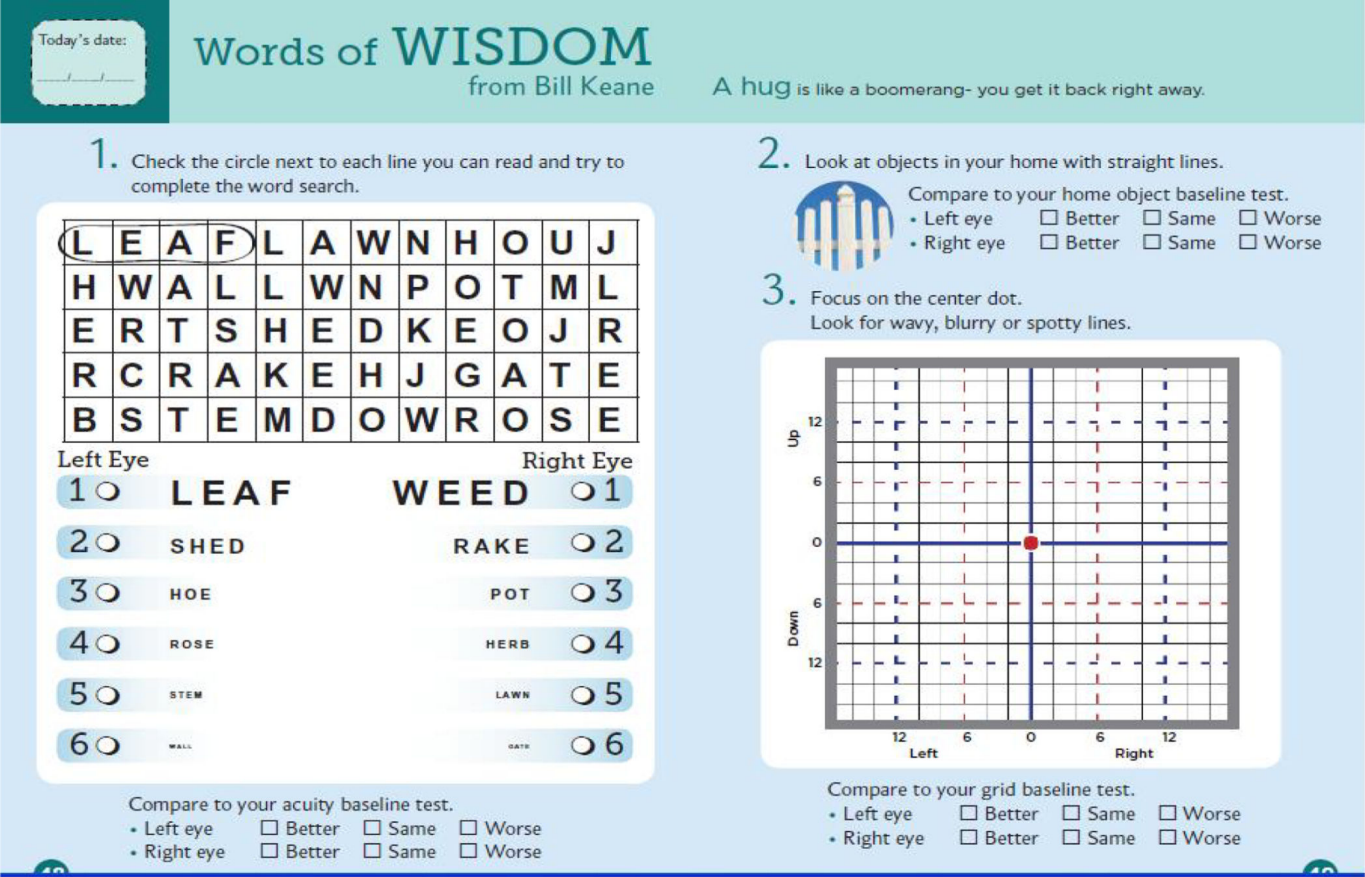
A set of weekly sample pages from the VMS journal.

The VMS journal contains an enhanced grid test with colored and dashed lines, but since an Amsler-type grid may not be sensitive for detecting new scotomatous areas, the VMS journal also includes a near visual acuity test and home objects reference test. Patients are encouraged to monitor their vision daily with objects in their environment, and to use the journal weekly to compare results against baseline. There are detailed instructions with diagrams on how to correctly use the tests and understand the results, as well as specific help-seeking steps to take if a change in vision is detected. A set of same pages from the VMS journal are presented in Figure 1. The VMS journal has been designed for distribution without the need for introduction or guidance by the doctor or office-staff, but ideally it could be presented to the patient at routine office visits.

The first goals of this randomized controlled trial (RCT) were to determine whether vision self-monitoring frequency and confidence were greater among intermediate stage, non-neovascular AMD patients who received the VMS journal compared to those receiving usual care (e.g. Amsler grid or instructions from their eye care provider). We also sought to determine whether the VMS journal would help promote adherence to weekly vision self-monitoring over the course of a year.

## Materials and Methods

### Subjects

The protocol for the study was approved by the Institutional Review Board of the Johns Hopkins University (JHU), School of Medicine and followed the tenets of the Declaration of Helsinki. Subjects were recruited from the retina division of the JHU Wilmer Eye Institute in Baltimore, Maryland, several retirement communities in Maryland, and retina specialists’ private practices in Connecticut (CT) and New York (NY). Following a review of eye exam records for eligibility, a total of 198 subjects were enrolled over the phone between January and December 2011 with an oral informed consent process explaining the nature and possible consequences of the study.

Subjects’ ocular disease status and corrected distance visual acuity (VA) were measured in the retinal specialists’ office using standard clinical tests at time of enrollment. Subjects with vision loss due to ocular pathology other than AMD or cataracts were excluded. Other exclusion criteria included cataract extraction in the last 3 months or capsulotomy in the last 24 hours in either eye, as well as those who were unable to give informed consent, non-English speaking or unable to complete the required study procedures.

### Study procedures

Randomization involved a 1:1 allocation and permuted blocks of random sizes that were stratified according to recruitment site. The sequence was generated by a secure computerized program and the randomization was provided via our database upon subject enrollment by our research assistants (ERA, AN and PB).

VMS journals were mailed to participants in the experimental group, with no training or education provided by the eye care provider or their staff. A <5 minute duration follow-up call occurred 2 weeks after the study materials were mailed to participants to confirm receipt of journal and address questions. No additional follow-up related to the VMS journal occurred after this initial contact, and no further phone contact following enrollment was provided to the subjects in the usual care control group during the 12 month follow-up period, other than to collect the 6 and 12 month follow-up questionnaire data.

The 6 and 12 month follow-up questionnaires were either completed by phone interviews by co-authors ERA, AN and PB or were self-completed by the participants via paper questionnaires at the time of the 6 and 12 month visits with the eye care provider. All calls to complete the questionnaires were made within 1 or 2 weeks of the 6 and 12 month follow-up visits with the eye care provider. The questionnaires included the 4-item Perceived Stress Scale [10], and also inquired about frequency of vision self-monitoring and confidence that they were taking care of their sight.

### Data analysis

The relationship between dichotomous variables, such as randomized group assignment versus frequency of vision monitoring, confidence in self-monitoring, weekly vision self-monitoring, gender, or AMD type, was assessed by Pearson’s chi squared (χ2) tests. AMD type was classified as either: (1) a previous episode of neovascular (NV) AMD in one eye and AREDS grade 3 or 4 non-neovascular AMD in the fellow eye, (2) AREDS grade 3 or 4 non-neovascular AMD (intermediate stage) in one eye and either AREDS grade 1 or 2 AMD (early stage) or no AMD in the fellow eye, or (3) AREDS grade 3 or 4 non-neovascular AMD (intermediate stage) in both eyes. Differences in continuous variables (e.g. age, VA) among the two randomized groups were examined by two sample t-tests. Multiple logistic regression models were used to explore factors that were predictors of weekly vision self-monitoring behavior and non-confidence in their vision monitoring. Data were analyzed using Stata/IC version 10.0 (Stata Corp., College Station, TX).

## Results

**Figure 2:**
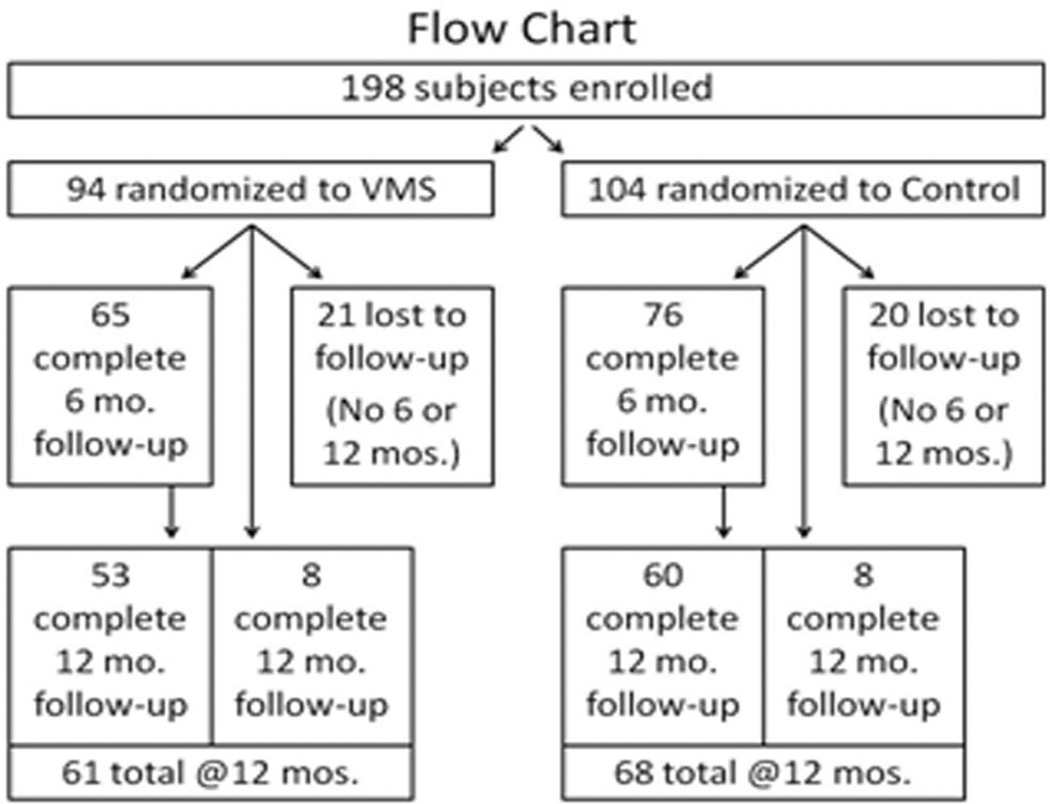
Diagram depicting the number of subjects at each follow-up time by randomized group.

Figure 2 is a flow chart with the number of subjects completing the 6 and 12 month follow-up questionnaires for each randomized group. Of the 198 participants, 94 were randomized to receive the VMS journal and 104 received usual care as the control group. Twenty-one subjects in the VMS journal group and 20 in the control group were lost to follow-up or developed neovascular AMD; i.e. they did not complete either the 6 or 12 month follow-up questionnaire. Note that a small proportion of subjects in each group completed the 12-month follow-up after missing the 6-month follow-up.

Approximately a fifth of the subjects (20.7%) were lost to follow-up, for the following reasons: 1.5% were deceased (N=3; n=1 VMS and n=2 controls), 9.6% developed physical illness or cognitive loss (N=19; n=9 VMS and n=10 controls), 5% were no longer interested in participating (N=10; n=5 VMS and n=5 controls), 3.5% were not reachable after several phone calls and a letter (N=7; n=5 VMS and n=2 controls), and 1% developed neovascular AMD prior to completing the 6 month follow-up (N=2; n=1 VMS and n=1 control).

The characteristics for each randomized group and all subjects who completed at least one follow-up are listed in Table 1 for age, baseline VA in the better seeing eye, baseline VA in the worse seeing eye, gender, AMD type and Perceived Stress Scale scores at 6 and 12 months. All except five subjects (one African-American, two Hispanics and two of mixed race) were identified as Caucasian. There were no statistically significant differences between the two groups (VMS vs. control) in terms of gender, type of AMD or Perceived Stress Scale scores. There was a statistically significant difference of 2.8 years (95% CI: 0.009, 5.6) in mean age, which may not be clinically meaningful in a population with a wide range of ages (49 to 96 years). Also, when compared to the subjects randomized to the control group, the subjects with the VMS journal had statistically significantly better VA on average in both the better and worse seeing eyes by 0.06 and 0.13 log units, respectively.

The questionnaire responses were obtained before the patient’s visit with the eye care provider in 65% of subjects, and after the eye care provider visit for 35% of subjects, which did not statistically significantly vary according to group randomization (χ^2^=0.02; p=0.88). The questionnaires were administered by phone to two-thirds of subjects, and in the eye care provider’s office for a third of the subjects, which did not statistically significantly vary according to group randomization (χ^2^=0.004; p=0.95). Weekly vision self-monitoring frequency was not significantly predicted by questionnaire administration mode (χ^2^=0.64; p=0.43) or timing relative to their eye exam (χ^2^=0.12; p=0.73).

**Figure 3:**
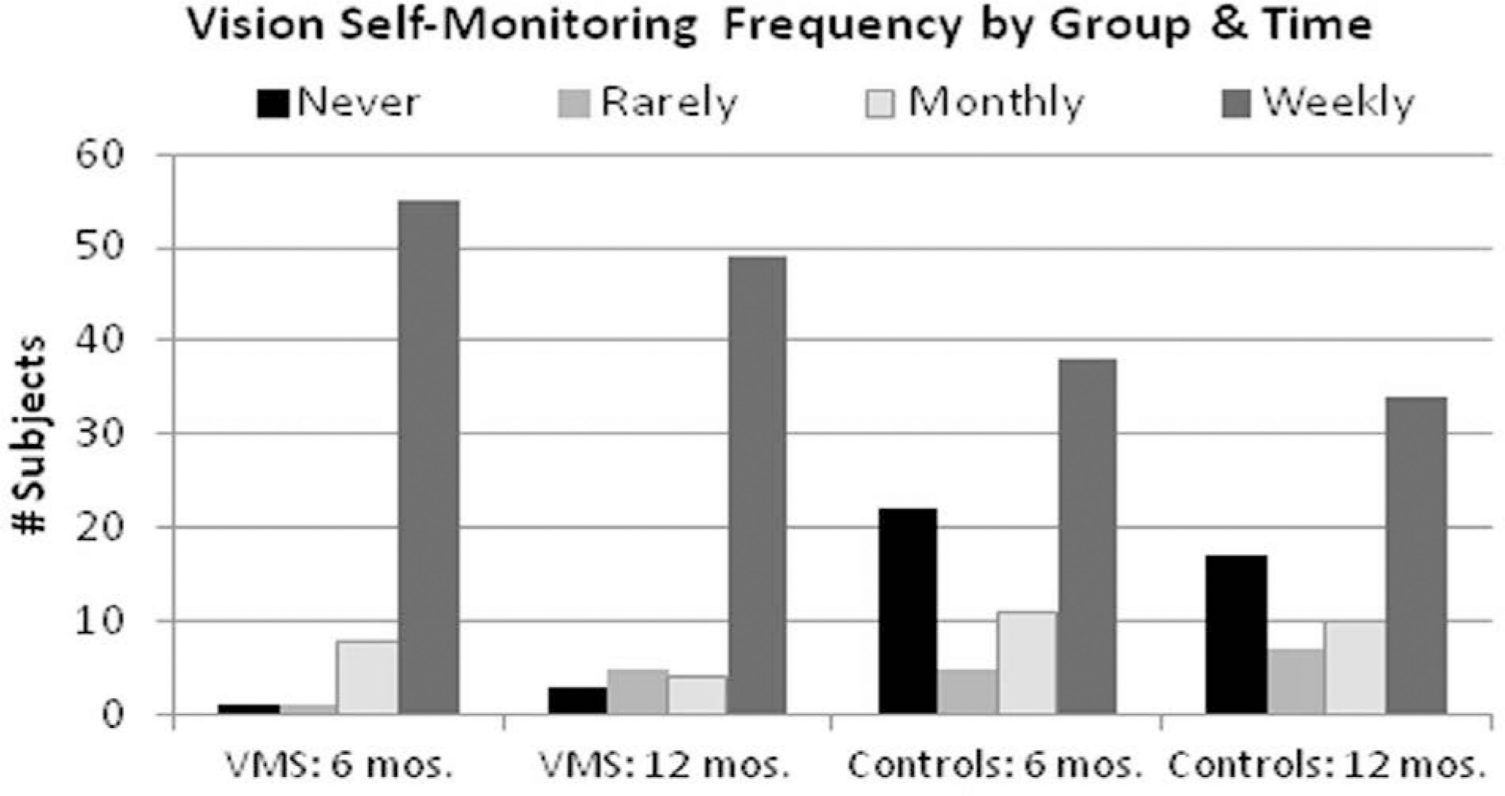
Self-reported frequency of vision self-monitoring at the 6 and 12 month follow-up evaluations for the subjects in the VMS journal group and usual care control group.

Figure 3 displays the subjects’ reported frequency of vision monitoring according to randomized group assignment. At 6 and 12 months, respectively, 29% and 25% of the control subjects (n=22 and 17) indicated that they had not checked their vision in the past 6 months, while 1.5% and 5% (n=1 and 3) of the subjects with the VMS journal reported that they did not check their vision. There was a statistically significant difference in the proportion of subjects in each group who reported vision monitoring at least weekly at 6 and 12 months, respectively: 85% and 80% of the subjects with the VMS journal vs. 50% of the control group at both follow-up times (p<0.001). Table 2a displays the multiple logistic regression model results for weekly vision self-monitoring, after adjusting for all other characteristic variables. Subjects with the VMS journal had statistically significant 7.1 and 4.2 times greater odds of reporting they self-monitored their vision weekly at 6 and 12 months, respectively, after adjusting for other variables. Gender, VA, AMD type, and perceived stress were not significantly related to weekly monitoring, but subjects recruited from the private retina practices in CT and NY were significantly more likely to monitor their vision weekly at the 6 month follow-up.

There was a highly statistically significant difference in the proportion of subjects who reported that they were not confident that monitoring their vision was helping to take care of their sight when comparing the VMS journal group to the usual care control group: 15% vs. 53% at 6 months, and 13% vs. 44% at 12 months (p<0.001). Table 2b displays the multiple logistic regression model results for non-confidence in self-monitoring, after adjusting for all other characteristic variables. Subjects in the usual care group had statistically significant 6.7 and 5.0 times greater odds of reporting non-confidence at 6 and 12 months, respectively, after adjusting for other variables. Gender, VA, AMD type, and perceived stress were not significantly related to non-confidence in vision self-monitoring. Older subjects were significantly more likely to report non-confidence at 12 months, but not 6 months, after adjusting for all other variables.

**Figure 4:**
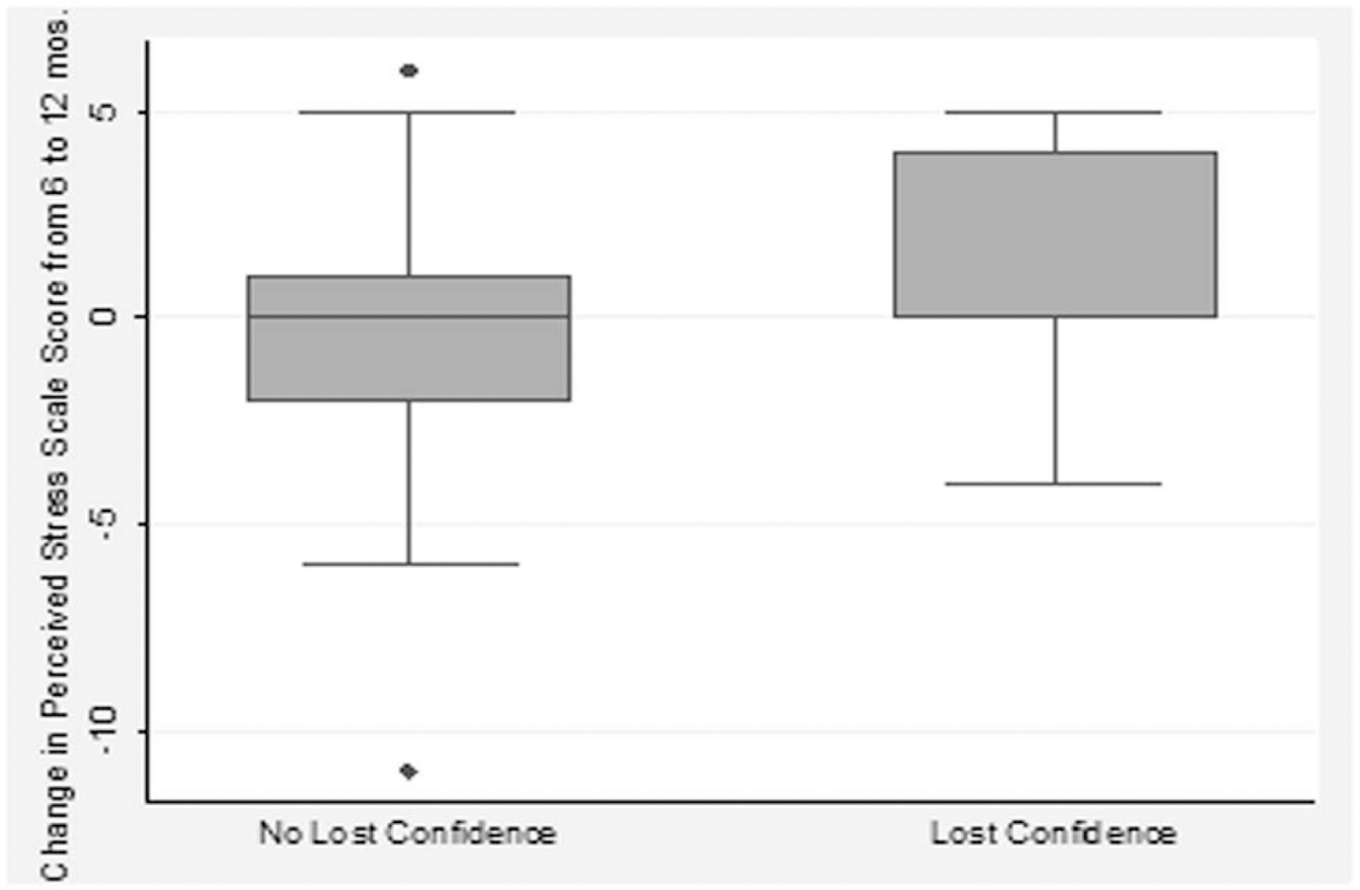
Box plot for changes in the Perceived Stress Scale score from 6 to 12 months according to subjects who did or did not develop a loss of confidence in their vision self-monitoring between 6 to 12 months. The bottom and top of the box are the 25^th^ and 75^th^ percentile (lower and upper quartiles, respectively), and the band near the middle of the box is the 50^th^ percentile (the median). Outliers by more than 1.5 times the interquartile range are indicated by dots.

Seventy-two percent of subjects (N=113; n=53 in VMS group and n=60 controls) completed both the 6 and 12 month questionnaires. We performed subgroup analyses of these 113 subjects to evaluate changes in responses over time from 6 to 12 months. There was no statistically significant change in weekly vs. less frequent self-monitoring between the groups (p=0.68), with 82% and 80% of the VMS group and control subjects, respectively, reporting no change in their frequency between 6 and 12 months. Only 13% of all subjects lost confidence from 6 and 12 months, which was not statistically significantly related to group assignment (p=0.54), but was significantly related to within-subject increases in Perceived Stress Scale scores from 6 to 12 months (p=0.03), shown in Figure 4. There was no significant relationship between changes in weekly monitoring frequency and changes in confidence from 6 to 12 months (p=0.74).

## Discussion

This RCT demonstrated that the VMS journal is successful at improving adherence to vision self-monitoring over a 12 month period and increasing subjects’ confidence in their ability to take care of their vision, when compared to usual care. A large majority of subjects randomized to receive the VMS journal reported checking their vision as least weekly, while only half of the usual care controls monitored their vision at least weekly. Roughly a quarter to a third of the usual care controls reported they did not monitor their vision at all in the past 6 months, whereas no more than 5% of the subjects with the VMS journal reported this. Only a small proportion of the group with the VMS journal reported a lack of confidence in vision self-monitoring, while approximately half of the usual care controls indicated that they did not have confidence.

There are other methods currently being studied to aid in the detection of new-onset neovascular AMD. Preferential hyperacuity perimetry (PHP) technology utilizes computer software to detect pathologic distortions indicating an early conversion to neovascular AMD [11,12]. A home monitoring device based on hyperacuity, the ForeseeHome [13], was tested in a large scale randomized controlled trial and found significantly better visual acuity after the development of neovacularization in subjects who used the device compared to usual care [14]. However, the expense incurred to monitor vision with the ForeseeHome device may well prohibit widespread use. A handheld shape discrimination hyperacuity test has been developed to self-monitor changes in maculopathy using a mobile device (iPhone app); however, future longitudinal studies are still needed to establish the thresholds for detecting clinically significant changes in shape discrimination hyperacuity in AMD patients who develop neovascularization, and to evaluate the sensitivity and specificity of the test to detect clinically important changes [15]. Two studies evaluating a 3D computer-automated threshold Amsler grid test (3D-CTAG) found that it may be more effective than the Amsler grid for detecting scotomas in non-neovasular or neovascular AMD patients [16,17]. However, research evaluating the 3D-CTAG has not yet demonstrated its ability to detect non-neovascular to neovascular AMD conversions or whether patients are capable of self-administering this test at home. These new tools may provide an elevated level of monitoring for some patients at high risk of converting to CNV. However, they do not address the large unmet need of many patients who either cannot afford such tools, do not have the desire or capability to adopt new technology, or are not yet considered high risk by their eye care provider. It is also likely that the cost and/or learning curve of new electronic technologies will limit broad-scale distribution, and a low-tech, low cost option, such as the VMS booklet, is necessary to reach large numbers of at-risk AMD patients.

A solution for AMD self-monitoring is still required that: (1) addresses the multiple reasons for patients’ delay in presenting for evaluation following new-onset neovascular AMD, (2) is low-cost and amenable to distribution across large populations, (3) includes multiple interactive elements to enhance compliance, and (4) fosters appropriate and timely action. We anticipate that the VMS journal fulfills these criteria and provides eye care providers with an easy to distribute alternative to the Amsler grid that reinforces their verbal instructions and improves their AMD patients’ self-management skills.

**Figure 5:**
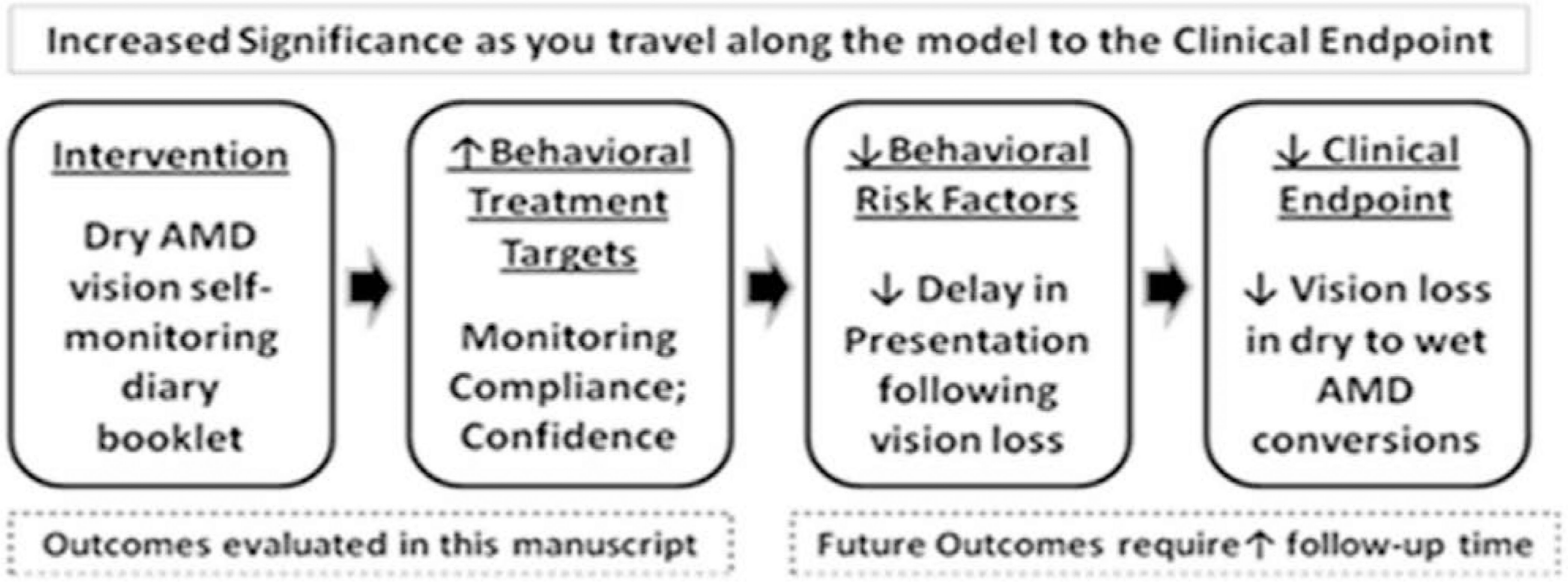
Conceptual model depicting the steps by which the VMS journal intervention is intended to promote behavior change and ultimately impact clinical outcomes.

The aim of the current study was to evaluate a minimal cost tool that can be provided to patients, in order to promote and enhance vision self-monitoring behavior in patients with high risk non-neovascular AMD. This is an important first step needed to address the goals of reducing: (1) the time it takes these patients to present to an eye care provider following true changes in vision indicative of newly developed neovascular AMD, and (2) the magnitude of vision loss following conversion to neovascular AMD. These steps to promote behavior change and ultimately impact clinical outcomes are shown in Figure 5. The RCT results thus far have shown that the VMS journal helps to increase vision monitoring behavior and patient confidence, which comprise a critical intermediate endpoint to help prevent the adverse clinical outcome of VA loss. Currently, the number of participants in our RCT who developed neovascular AMD or >3 lines of VA loss is insufficient (i.e. ≤5 subjects) to form conclusions about the efficacy of the VMS to prevent vision loss. Continued study with the recruitment of additional subjects and/or longer-term monitoring of participants in the RCT should help determine the efficacy to achieve clinical endpoints.

The VMS journal intervention included a few minimal yet additional, inherent components that were not provided to usual care controls (i.e. receiving the journal in the mail, then a follow-up phone call to ensure receipt and understanding) since we were interested in comparing the VMS journal intervention to typical clinical practice. To meet our large recruitment goal, it was also not feasible to perform informed consent, randomize and distribute the VMS journal in the eye care providers offices at the same time when usual care (Amsler grid) was provided. While it is possible that some of our effects could have been due in part to this difference in the interventions, we believe this is not likely a significant factor since both groups received equal contact between the 6 and 12 month follow-ups and significant differences persisted.

There are other demographic, socioeconomic and general health factors (e.g. income, education level, systemic comorbidities) that could have influenced our outcomes, but these demographic data were not collected in the present study since we had no reason to expect that these factors or their effects would be significantly different between our two groups after randomization. AMD severity, i.e. VA and monocular versus binocular disease, did not significantly influence our outcomes.

Our loss to follow-up rate of 21% was not significantly different between groups, and is comparable to other RCTs with AMD patients that did not involve surgical interventions or intravitreal injections. Two previous RCTs involving nutritional supplements in AMD demonstrated a 15–20% loss to follow-up rate at 12 months [18,19], and another trial of lutein supplementation in AMD had a 20% dropout rate by 3 months [20]. More than half of the losses to follow-up in our study were associated with age-related health factors or issues: 1.5% were deceased (N=3; n=1 VMS and n=2 controls), 9.6% developed physical illness or cognitive loss (N=19; n=9 VMS and n=10 controls), and 1% developed neovascular AMD prior to completing the 6 month follow-up (N=2; n=1 VMS and n=1 control). Only 3.5% were unreachable after several phone calls and a letter (N=7; n=5 VMS and n=2 controls). Five percent of participants stated that they were no longer interested in participating (N=10; n=5 VMS and n=5 controls). In future studies, the loss to follow-up rate may be reduced by asking participants during informed consent whether they would like to name a family member or friend as an alternative contact to help locate participants who fail to respond to phone calls or a letter. More frequent contact with participants would likely reduce loss to follow-up; however, we were interested in testing our intervention similarly to how it would be implemented outside of a study, i.e., with patients being advised to monitor their vision independently, without reminders.

A strength of our study was recruitment from various sources in the northeastern United States, thus enhancing the generalizability of the results to similar populations attending academic medical centers or retinal specialists’ private practices for ophthalmic care, as well as to individuals residing in retirement communities in this region. Our finding that subjects from the retina specialists’ offices in CT and NY were significantly more likely to monitor their vision weekly suggests that the providers at these private practices may place greater emphasis on promoting self-monitoring with the Amsler grid than the retina specialists at an academic medical institution (JHU).

In conclusion, this RCT demonstrated the ability of the VMS journal to promote increased vision self-monitoring frequency and confidence among patients with intermediate non-neovascular AMD and maintain high adherence over a one-year period. This low-cost, easy to administer intervention has high potential for mass distribution and broad impact. Our ongoing research aims to demonstrate that this fundamental behavior change of increased vision self-monitoring frequency and confidence, coupled with the VMS journal’s clear, repetitive and consistent educational messages, will serve as a catalyst to overcome reasons for patient delay and reduce presentation time to the eye care provider after the development of vision changes due to neovascularization in AMD.

## Figures and Tables

**Table 1:** Characteristics of each randomized group and the total study population.

	VMS journal group		Usual care Control group		All Subjects (N=157)		
**Continuous variables**	**mean (range)**	**SD**	**mean (range)**	**SD**	**mean (range)**	**SD**	**P-value**
Age (years)	74.0 (53,96)	8.9	76.8 (49,93)	8.7	75.5 (49,96)	8.9	0.049*
VA better eye (logMAR)	0.15 (−0.1,0.56)	0.12	0.21 (−0.05,1.1)	0.21	0.18 (−0.1,1.1)	0.18	0.04*
VA worse eye (logMAR)	0.32 (0.0,1.6)	0.30	0.45 (0.0,~1.6)	0.38	0.39 (0.0, ~1.6)	0.35	0.03*
Perceived Stress @ 6mos.	3.37 (0,10)	2.54	3.46 (0,14)	3.04	3.42 (0,14)	2.81	0.85
Perceived Stress @ 12mos.	2.77 (0,13)	2.59	3.12 (0,12)	2.95	2.95 (0,13)	2.78	0.48
**Dichotomous variables**	**Number (%)**		**Number (%)**		**Number (%)**	**χ^2^;**	**P-value**
Female Gender	48 (65.8%)		44 (52.4%)		92 (58.6%)	2.9	0.09
AMD type							
previous NV AMD one eye	9 (12.3%)		11 (13.1%)		20 (12.7%)		
intermediate AMD one eye	21 (28.8%)		24 (28.6%)		45 (28.7%)		
intermediate AMD both eyes	43 (58.9%)		49 (58.3%)		92 (58.6%)		

* Statistically significant (p<0.05)

**Table 2:** Adjusted^a^ odds of subjects reporting vision self-monitoring at least weekly at 6 or 12 months. b. Adjusted^a^ odds of subjects reporting no confidence in vision self-monitoring at 6 or 12 months.

	6 month follow-up			12 month follow-up		
**a. Weekly vision self-monitoring**	**OR**	**95% CI**	**P-value**	**OR**	**95% CI**	**P-value**
VMS group vs. Control group	7.12	(2.68,18.9)	<0.001*	4.18	(1.68,10.4)	0.002*
Age (years)	0.97	(0.91,1.02)	0.22	0.96	(0.91,1.02)	0.19
Female Gender	1.00	(0.40,2.5)	0.99	1.35	(0.53,3.4)	0.53
VA better eye (logMAR)	0.75	(0.03,16.8)	0.85	2.02	(0.07,62.7)	0.69
VA worse eye (logMAR)	0.39	(0.07,2.2)	0.28	1.21	(0.21,6.8)	0.83
AMD type (intermediate both eyes)	1.27	(0.32,5.1)	0.73	1.31	(0.35,4.9)	0.69
AMD type (intermediate one eye)	0.57	(0.11,2.9)	0.50	1.30	(0.26,6.5)	0.75
Perceived Stress Scale Score	1.13	(0.95,1.35)	0.18	0.94	(0.80,1.11)	0.48
NY/CT Retina Specialists vs. JHU	4.69	(1.5,14.3)	0.007*	2.42	(0.85,6.9)	0.10
Retirement communities vs. JHU	6.58	(0.68,63.4)	0.10	3.75	(0.39,35.8)	0.25
**b. No confidence in self-monitoring**	**OR**	**95% CI**	**P-value**	**OR**	**95% CI**	**P-value**
VMS group vs. Control group	0.15	(0.06,0.38)	<0.001*	0.20	(0.07,0.56)	0.002*
Age (years)	1.03	(0.98,1.09)	0.27	1.09	(1.02,1.16)	0.016*
Female Gender	1.35	(0.56,3.22)	0.51	0.99	(0.37,2.7)	0.997
VA better eye	0.90	(0.05,17.02)	0.94	8.24	(0.22,308.4)	0.25
VA worse eye	1.33	(0.28,6.4)	0.73	0.44	(0.07,2.9)	0.39
AMD type (intermediate both eyes)	2.41	(0.62,9.5)	0.21	0.44	(0.11,1.8)	0.26
AMD type (intermediate one eye)	2.32	(0.48,11.1)	0.29	0.99	(0.19,5.2)	1.0
Perceived stress scale score	0.90	(0.77,1.05)	0.18	1.14	(0.96,1.36)	0.14
NY/CT Retina Specialists vs. JHU	0.58	(0.21,1.62)	0.30	0.92	(0.28,2.96)	0.88
Retirement communities vs. JHU	0.29	(0.04,2.4)	0.25	0.35	(0.03,3.9)	0.40

a Adjusted model includes all variables

* Statistically significant (p<0.05)
